# Poor health-related quality of life in postural orthostatic tachycardia syndrome in comparison with a sex- and age-matched normative population

**DOI:** 10.1007/s10286-023-00955-9

**Published:** 2023-06-20

**Authors:** Marie-Claire Seeley, Celine Gallagher, Eric Ong, Amy Langdon, Jonathan Chieng, Danielle Bailey, Annabelle Dennis, Nikki McCaffrey, Dennis H. Lau

**Affiliations:** 1 Australian Dysautonomia and Arrhythmia Research Collaborative, The University of Adelaide, Adelaide, Australia; 2 South Australian Health and Medical Research Institute, Adelaide, SA Australia; 3 College of Medicine and Public Health, Flinders University, Adelaide, SA Australia; 4 Deakin Health Economics, Institute for Health Transformation, School of Health and Social Development, Faculty of Health, Deakin University, Melbourne, VIC Australia; 5 Department of Cardiology, Royal Adelaide Hospital, 1 Port Road, Adelaide, SA 5000 Australia

**Keywords:** Postural orthostatic tachycardia syndrome, Health-related quality of life, Health utility, Orthostatic intolerance, Global health rating

## Abstract

**Purpose:**

The effect of postural orthostatic tachycardia syndrome (POTS) on health-related quality of life (HrQoL) remains poorly studied. Here, we sought to compare the HrQoL in individuals with POTS to a normative age-/sex-matched population.

**Methods:**

Participants enrolled in the Australian POTS registry between 5 August 2021 and 30 June 2022 were compared with propensity-matched local normative population data from the South Australian Health Omnibus Survey. The EQ-5D-5L instrument was used to assess HrQoL across the five domains (mobility, self-care, usual activities, pain/discomfort, and anxiety/depression) with global health rating assessed with a visual analog scale (EQ-VAS). A population-based scoring algorithm was applied to the EQ-5D-5L data to calculate utility scores. Hierarchical multiple regression analyses were undertaken to explore predictors of low utility scores.

**Results:**

A total of 404 participants (*n* = 202 POTS; *n* = 202 normative population; median age 28 years, 90.6% females) were included. Compared with the normative population, the POTS cohort demonstrated significantly higher burden of impairment across all EQ-5D-5L domains (all *P* < 0.001), lower median EQ-VAS (*p* < 0.001), and lower utility scores (*p* < .001). The lower EQ-VAS and utility scores in the POTS cohort were universal in all age groups. Severity of orthostatic intolerance symptoms, female sex, fatigue scores, and comorbid diagnosis of myalgic encephalomyelitis/chronic fatigue syndrome were independent predictors of reduced HrQoL in POTS. The disutility in those with POTS was lower than many chronic health conditions.

**Conclusions:**

This is the first study to demonstrate significant impairment across all subdomains of EQ-5D-5L HrQoL in the POTS cohort as compared with a normative population.

**Trial registration:**

ACTRN12621001034820

**Supplementary Information:**

The online version contains supplementary material available at 10.1007/s10286-023-00955-9.

## Introduction

Postural orthostatic tachycardia syndrome (POTS) is an autonomic disorder identified by its hallmark manifestation of postural-induced tachycardia in the absence of orthostatic hypotension [1, 2]. The syndrome has a high female predominance (> 80%) and appears to mostly affect those of childbearing age [3–5]. Patients with POTS often experience diverse and multisystemic symptomatology with variable impact on activities of daily living including ability to self-care or engage in education and employment [6]. POTS remains poorly recognized among clinicians, with reports of protracted delay in diagnosis [5, 6]. The impact of this condition has not been well characterized to date, with few studies reporting on health-related quality of life (HrQoL) in those affected [7, 8]. Furthermore, the effect of POTS on HrQoL, including restriction of activity and societal participation in comparison with normative age-matched populations, has not been evaluated. The lack of such information prevents healthcare agencies from understanding the true impact of POTS and limits their ability to allocate health resources equitably. Therefore, the primary objective of this study was to delineate the disutility of POTS in comparison with a normative age- and sex-matched population.

The Euroquol five-dimensional instrument (EQ-5D) is the most ubiquitously applied generic multi-attribute utility instrument for indirect measurement of health utilities by assessing five domains including mobility, self-care, usual activities, pain/discomfort, and anxiety/depression, as well as the global health status in an individual [9–11]. Here, we used the contemporary five-response version of the Euroquol tool (EQ-5D-5L), which has demonstrated strong sensitivity in detecting clinically meaningful differences in HrQoL, to evaluate the health status of patients with POTS. These were compared with local normative age- and sex-matched data from participants in the South Australian Health Omnibus Study [10].

## Methods

This study has institutional human research and ethics committee approval (approval number H-2021–052) and was performed in accordance with the 1964 Declaration of Helsinki standards for human research. All participants gave informed consent prior to their inclusion in the study.

Consecutive participants ≥ 16 years of age and enrolled in the Australian POTS patient registry (Australian New Zealand Clinical Trials Registry: ACTRN12621001034820) between 5 August 2021 and 30 June 2022, who provided written, informed consent were included. Health and quality of life surveys, including the composite autonomic symptom score (COMPASS-31) and fatigue severity (FSS) were completed via a password protected, electronic link to a Research Electronic Data Capture (REDCap) database. The REDCap self-complete digital version of the EQ-5D-5L was utilized to capture quality of life responses. To be eligible, all participants had their POTS diagnosed or confirmed by an experienced multidisciplinary team at a specialist clinic in Adelaide, South Australia, according to the international criteria, which included a sustained heart rate rise of ≥ 30 beats per minute (bpm) (≥ 40 bpm in adolescents) on 10 min standing test or head-up tilt table; absence of orthostatic hypotension as defined by a drop of ≥ 20 mmHg systolic or 10 mmHg diastolic blood pressure in the first 3 minutes of standing; as well as accompanying symptoms of orthostatic intolerance and chronicity of symptomology for ≥ 6 months [2]. All comorbidities were clinically verified and most of the individuals with POTS were treatment naive. Data were compared with those of local normative age- and sex-matched participants from the South Australian Health Omnibus Study for this comparative study. In brief, this database consists of 2908 individuals randomly selected from both metropolitan Adelaide and country towns with population of at least 1000 people. The interviewer-administered, face-to-face, cross-sectional study was undertaken in 2013 where deidentified sociodemographic and EQ-5D-5L responses were collected [10].

### Health-related quality of life measure

The EQ-5D was developed in 1990 and is now the world's most ubiquitously applied generic multi-attribute utility instrument for indirect measurement of health utilities [12–15]. The contemporary five-response version (EQ-5D-5L) was used as it has shown improved convergence validity and less potential for ceiling effects than its three-response predecessor [9, 10, 12, 16]. The EQ-5D-5L demonstrates strong sensitivity in detecting clinically meaningful differences in HrQoL and can be utilized as a nondisease-specific assessment tool for analysis of health status. The descriptive instrument assesses responses across five domains: mobility, self-care, usual activities, pain/discomfort, and anxiety/depression, using a five-response Likert scale. Each dimension of the survey has five responses ranging from no problems (1) through to extreme problems (5). A total of 3125 possible health states exists and range from full health (11,111) to worst health (55,555) [10]. Further, a visual analog scale (EQ-VAS) is used to solicit a global health scale from respondents on a 0–100 scale, with 100 equals to a state of full health.

Disutility is a health economic term used to describe the reduction in valued HrQoL caused by differing health conditions and can be used to compare the relative impact of varying health conditions and inform allocation of health resources [9, 10, 12, 16]. HrQoL measures are commonly represented by quality weights (utilities), which are anchored on a scale from 0 to 1, where 0 equals a state commensurate with death and 1 equal full health. When a health state is considered “worse than death,” the value may be negative. In the absence of an Australian value sets, the Devlin et al. (2016) UK dataset was utilized to apply a scoring algorithm to the EQ-5D-5L data to calculate utility scores in the POTS cohort, as was previously applied to the normative dataset from the South Australian Health Omnibus Study to facilitate comparison[9, 10].

### Statistical analysis

Continuous data are expressed as mean and standard deviation or median and interquartile range (IQR) according to distribution. Frequencies and percentages were used for categorical variables. For continuous variables, the Mann–Whitney *U*-test (two groups) and Kruskal–Wallis one-way analysis of variance (multiple groups) were used, while the Chi-squared test was used for categorical data. Propensity matching according to age and sex was undertaken using the MatchIt package in R, version 4.1.2. Hierarchical multiple regression analyses were undertaken to explore potential predictors of low utility scores with a minimally adjusted model to account for variables previously reported to influence HrQoL, including age and sex. [10, 17] The following comorbidities (anxiety, asthma, autism spectrum disorder, celiac disease, depression, endometriosis, hypermobile Ehlers–Danlos syndrome or hypermobility spectrum disorder, irritable bowel syndrome, fibromyalgia, migraine, myalgic encephalitis or chronic fatigue syndrome, and polycystic ovary disease), FSS total score, and COMPASS-31 measures of autonomic dysfunction were utilized in the fully adjusted model. All data were analyzed using SPSS statistics (version 28.0, IBM Inc, Armonk, NY, USA) and statistical significance was set at *P* < 0.05.

## Results

From a total of 256 participants enrolled in the Australian POTS patient registry during the study period, 54 were excluded due to incomplete surveys. This study therefore included 202 consecutive participants with POTS and 202 age and sex propensity matched normative participants. The median age of these patients was 28 years (range 16–66 years). The majority of the participants were female (90.6%) and Caucasian (95%). POTS patients were less likely to be undertaking any employment (47% versus 62%, *P* < 0.001) and more likely to be unemployed due to disability or injury (15% versus 4%, *P* < 0.001) than the normative population. The POTS cohort reported onset of POTS symptoms at median age of 23 years (IQR 16 years) with median symptom duration of 3 years (IQR 9 years). As presented in Table 1, 40% of the individuals with POTS demonstrate no identifiable trigger, while preceding infection was reported in 30%. Other less common triggers are trauma or concussion, post-surgical, and vaccination. Common comorbidities include anxiety or depression, migraine, Ehlers–Danlos syndrome or hypermobile spectrum disorder, irritable bowel syndrome, iron deficiency, and myalgic encephalomyelitis or chronic fatigue syndrome (Table 1).

**Table 1:** Characteristics of POTS cohort

	POTS *n* = 202	Matched cohort *n* = 202
Age (years), median (IQR)	28 (16)	28 (16)
Female sex, *n* (%)	90.6	90.6
Age of symptom onset (years), median (IQR)	23 (16)	
Caucasian race, *n* (%)	192 (95)	
POTS trigger, *n* (%)		
No identifiable cause	81 (40)	
Viral infection	55 (27)	
Post trauma/concussion	16 (8)	
Post vaccination	14 (7)	
Post-surgical	9 (4)	
Bacterial infection	8 (4)	
Post pregnancy	7 (3)	
Allergic reaction	3 (2)	
Eating disorder	3 (2)	
Other	6 (3)	
Comorbid diagnosis, *n* (%)		
Anxiety disorders	102 (50)	
Migraine	92 (45)	
Depression	77 (38)	
Ehlers–Danlos syndrome	74 (36)	
Hypermobile spectrum disorder	43 (21)	
Irritable bowel syndrome	70 (35)	
Iron deficiency	68 (34)	
Myalgic encephalomyelitis/chronic fatigue syndrome	57 (28)	
Fibromyalgia	46 (23)	
Asthma	44 (22)	
Endometriosis	40 (20)	
Polycystic ovarian disease	22 (11)	
Autism spectrum disorder	15 (7)	
Celiac disease	14 (7)	
	*n* = 202	
Age of symptom onset (years), median (IQR)	23 (16)	
Caucasian race, *n* (%)	192 (95)	
POTS trigger, *n* (%)		
No identifiable cause	81 (40)	
Viral infection	55 (27)	
Post trauma/concussion	16 (8)	
Post vaccination	14 (7)	
Post surgical	9 (4)	
Bacterial infection	8 (4)	
Post pregnancy	7 (3)	
Allergic reaction	3 (2)	
Eating disorder	3 (2)	
Other	6 (3)	
Comorbid diagnosis, *n* (%)		
Anxiety disorders	102 (50)	
Migraine	92 (45)	
Depression	77 (38)	
Ehlers–Danlos syndrome	74 (36)	
Hypermobile spectrum disorder	43 (21)	
Irritable bowel syndrome	70 (35)	
Iron deficiency	68 (34)	
Myalgic encephalomyelitis/chronic fatigue syndrome	57 (28)	
Fibromyalgia	46 (23)	
Asthma	44 (22)	
Endometriosis	40 (20)	
Polycystic ovarian disease	22 (11)	
Autism spectrum disorder	15 (7)	
Celiac disease	14 (7)	

### Health-related quality of life

**Fig. 1:**
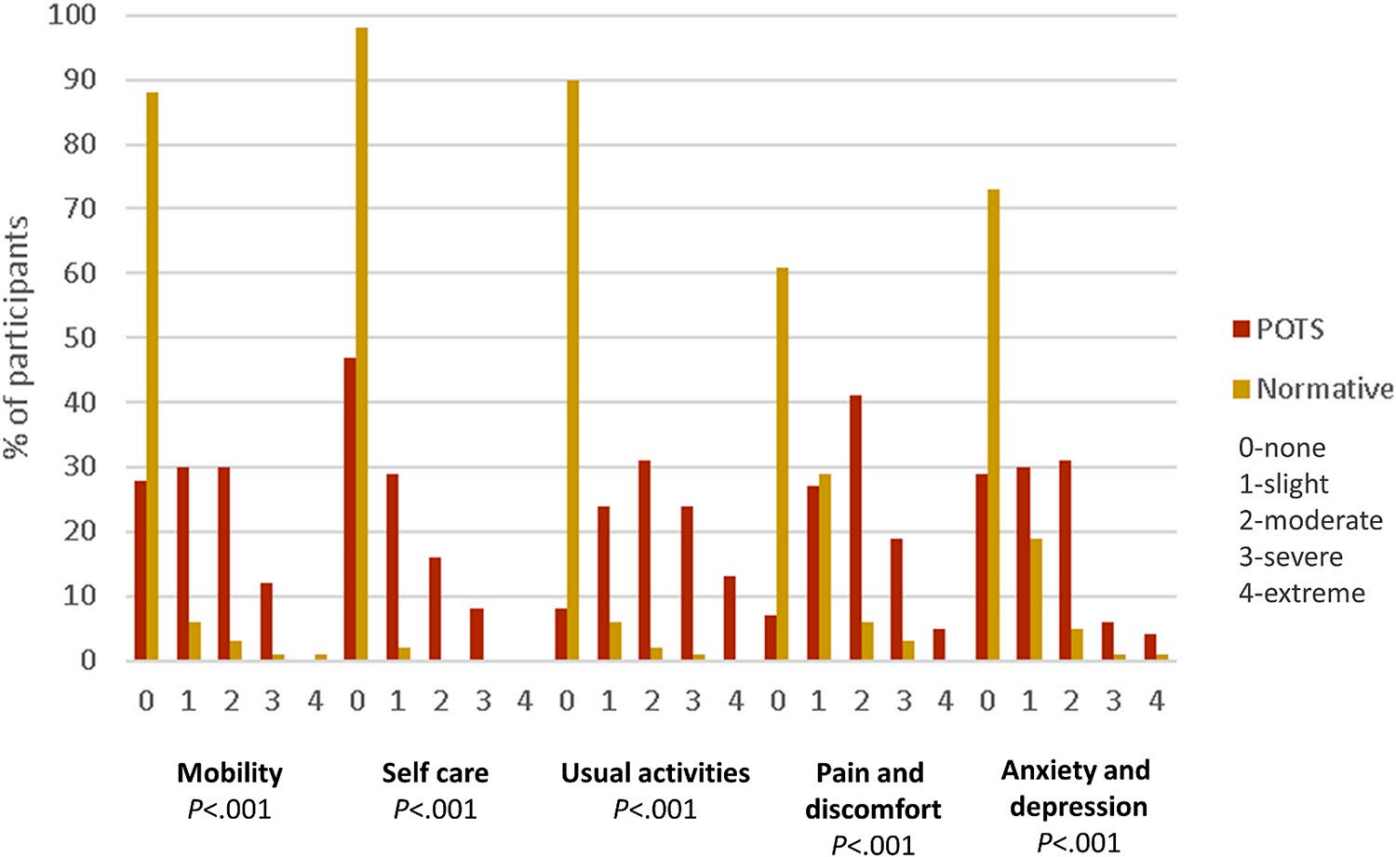
Comparative mean EQ-5D-5L score by subdomain

**Fig. 2:**
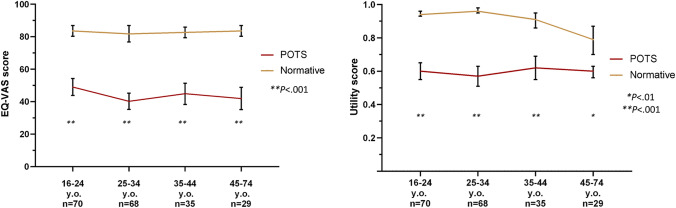
Comparative mean EQ-VAS and utility scores by age category

Individuals with POTS experienced significantly higher level of impairment across all subdomains of the EQ-5D-5L as compared with the normative population (all *P* < 0.001, Fig. 1 and Supplementary Table 1). Specifically, moderate-to-extreme restrictions were encountered most frequently with “usual activities” followed by “pain and discomfort,” “mobility,” “anxiety and depression,” and “self-care” in the POTS cohort (68%, 65%, 42%, 41%, and 24%, respectively). Notably, the prevalence of moderate-to-extreme restrictions in the corresponding subdomains were much lower in the normative population (4%, 10%, 6%, 8%, and 0%, respectively). Additionally, the median global health rating (EQ-VAS) was significantly lower in the POTS cohort as compared with the normative population [40 (IQR 30) versus 85 (IQR 25), *P* < 0.001]. Similarly, the calculated median utility score was significantly lower in the POTS cohort as compared with the normative population [0.63 (IQR 0.32) versus 0.95 (IQR 0.10), *P* < 0.001]. Notably, the lower EQ-VAS and utility scores in the POTS cohort were universal in all age groups including those in the younger age groups (16–24 and 25–34 years old, Fig. 2 and Supplementary Table 2).

### Predictors of poorer health related quality of life in POTS

Age and sex only explained 0.7% of variances, while the comorbidities and COMPASS-31 domain scores accounted for 33.7% variances in the calculated utility score (*P* < 0.001). Hierarchical multiple regression analyses found three significant predictors for lower utility score in the POTS cohort: severity of orthostatic intolerance symptoms from COMPASS-31 (*P* < 0.001), female sex (*P* < 0.02), and comorbid diagnosis of myalgic encephalomyelitis/chronic fatigue syndrome (*P* = 0.04). When exploring predictors of EQ-VAS, age and sex explained 3.4% of variances, while comorbidities, fatigue scores, and COMPASS-31 domains accounted for 32.6% of the change in scores (*P* < 0.001). Orthostatic intolerance (*P* < 0.001), female sex (*P* = 0.001), myalgic encephalomyelitis/chronic fatigue syndrome diagnosis (*P* = 0.01), and FSS (*P* = 0.02) were all statistically significant contributors to this hierarchical regression model.

## Discussion

This is the first study to report comparative HrQoL data in individuals with POTS and a normative, age- and sex-matched population. First, our data demonstrate new findings of significantly reduced HrQoL in the POTS cohort as compared with a normative population in the form of significantly lower EQ-5D-5L scores in all five subdomains, with “usual activities” being the worst affected, followed by “pain and discomfort,” “mobility,” “anxiety and depression,” and “self-care.” Second, individuals with POTS reported significantly lower global health rating and higher health disutility. Third, there is no sparing of these effects with similarly affected HrQoL scores among different age categories. Last, severity of orthostatic intolerance symptoms, female sex, and comorbid diagnosis of myalgic encephalomyelitis/chronic fatigue syndrome were independent predictors of lower utility scores. Our findings provide new understanding on the disutility associated with POTS and calls for improved health response to this condition.

### Significance of reduced health-related quality of life in POTS

Using the 36-item short-form health survey (SF-36), Benrud-Larson and colleagues reported significantly impaired physical functioning, role functioning, bodily pain, general health, vitality, and social functioning in 94 individuals with POTS, similar to that reported by patients with chronic obstructive pulmonary disease and congestive heart failure. Notably, they did not find any significant reduction in mental health or role limitations due to emotional problems in their POTS cohort, while our data showed significantly higher anxiety and depression scores using the EQ-5D-5L. Others have previously compared EQ-VAS scores between POTS (*n* = 44) and healthy control subjects (*n* = 46) and found similarly reduced global health rating among individuals with POTS (53 ± 17 versus 89 ± 7). [19] Previous work from a large number of individuals with self-reported physician-diagnosed POTS found no difference in RAND-36 overall health-related composite scores between male and female patients despite lower RAND-36 physical health-related composite scores in females and lower mental health-related composite scores in males. In contrast, we found female sex to be an independent predictor of lower health utility scores in our POTS population, which was in keeping with previously published normative population data from South Australia; however, the high representation of female sex in this POTS cohort (91%) may confound the generalizability of this finding.[10].

**Fig. 3:**
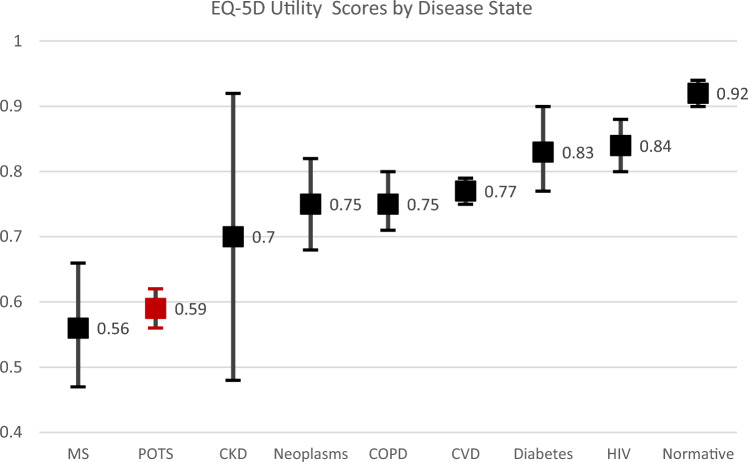
Mean EQ-5D-5L scores in comparison with other disease states as reported by Zhou et al. Mean score with 95% confidence interval. MS; multiple sclerosis, POTS; postural orthostatic tachycardia syndrome, CKD; chronic kidney disease, COPD, chronic obstructive pulmonary disease, CVD; cardiovascular disease, HIV; human immunodeficiency virus

There are multiple other studies that have utilized EQ-5D tools to assess HrQoL in different chronic conditions. One meta-analysis demonstrated a higher prevalence of extreme problems across all subdomains of the EQ-5D-3L in those with POTS compared with control or reference data [20]. Furthermore, a meta-analysis of 35 studies using EQ-5D-5L by Zhou and colleagues, explored health utility across seven diseases. The POTS cohort in this study demonstrated worse HrQoL compared with those reported for diabetes mellitus, neoplasms, cardiovascular disease, chronic obstructive pulmonary disease, human immunodeficiency virus infection, and chronic kidney disease [11]. The only health condition in the meta-analysis with lower HrQoL than our POTS cohort was multiple sclerosis (Fig. 3). The stark difference in HrQoL is particularly concerning when consideration is given to the relatively young age of the POTS population compared with most of these chronic diseases. Indeed, our data found that the impact of POTS on HrQoL was uniform across all age categories, with similarly low EQ-VAS and utility scores even in those ≤ 34 years of age.

The significant impact of POTS on HrQoL could perhaps be explained by the syndrome itself, in which diverse and multisystem symptomatology is common because of the many bodily functions such as gastrointestinal motility and digestion, genitourinary control, pupillary reflexes, salivary and sweat control, as well as blood pressure and heart rate control that could be affected by autonomic nervous system dysfunction. It is also not surprising that the presence of myalgic encephalomyelitis/chronic fatigue syndrome, as well as the severity of orthostatic intolerance, could contribute to lower utility and HrQoL as seen in our hierarchical multiple regression analyses. Previous studies have identified a high prevalence of autonomic dysfunction in those with myalgic encephalomyelitis/chronic fatigue syndrome as well as a predominance of fatigue symptoms among both these populations [21–23]. Additionally, others have shown strongest correlation between symptoms of orthostatic intolerance and the SF-36 physical component scale [8]. In another study of 107 patients with POTS, severity of orthostatic intolerance symptoms were significantly inversely correlated with both SF-36 physical and mental scales [7]. While these may represent markers of more severe POTS, further work is needed to delineate the pathophysiological links between myalgic encephalomyelitis/chronic fatigue syndrome and POTS [24]. It is noteworthy that in our cohort, orthostatic intolerance was the only autonomic subdomain statistically associated with reduced quality of life. It is possible that management of orthostatic intolerance symptoms may improve both fatigue and HrQoL, and clinicians could prioritize this aspect of care in the management of individuals with POTS.

### Impact of reduced health-related quality of life in POTS

The reduced HrQoL in the POTS cohort may result in interruptions to education, career, and lifespan development, resulting in reduced lifetime earning capacity, child raising capacity, and general detrimental psychosocial disadvantages [6, 17]. Outside of the experiences of the individuals with POTS, there is also the presumed effects on family members who, in the absence of societal health and disability support, are forced to bridge the gap as carers, advocates, and income earners. Increased healthcare utilization is well documented in individuals with POTS and may reflect the chronic debilitating nature of this condition.[6] Yet POTS has not been recognized as a disease entity, having no International Classification of Disease code until the most recent iteration was granted for use in the USA in October 2022. Elsewhere, POTS remains a “hidden” illness, with no viable way for health providers to track the health utilization and burden of the disease through disease coding. In practice, the absence of a unique code creates an added disincentive for the healthcare sector to provide treatment options for this condition, both in the private and socialized settings. The lack of recognition of the high disability experienced by those with POTS, and the lasting impacts on educational and employment absenteeism, has likewise limited access to disability support from funding agencies. Our finding of significantly lower utility scores in the POTS cohort adds to the limited pool of evidence regarding the low HrQoL experienced by those affected, and the comparison of the utility score to other chronic diseases provides an important reference regarding the impact of this condition.

### Study limitations

There are several limitations to this study. Our data stem from a single cohort of individuals with POTS in Australia at a single timepoint. Additional longitudinal data from multiple centers would help strengthen the evidence on HrQoL in POTS and its course over time with ongoing treatment. It is likely that the more severely affected or symptomatic individuals with POTS were unintentionally excluded from this study as they are often the ones who could not complete all the online surveys for the registry. We recognize that the EQ-5D-5L was undertaken at different time periods between the normative population (2013) and the POTS cohort (2021–2022) and there may be unknown external confounders that may have impacted the results. It is unclear how the SARS-CoV-2 pandemic during the study period would have impacted on the HrQoL scores in our POTS cohort, although our scores were similar to previous study using the same EQ-5D-5L tool [18]. Further, the data on the comorbidities or medical diagnosis of the normative population were not collected and are therefore unavailable for comparison.

## Conclusions

This is the first comprehensive comparison of HrQoL in POTS with an age- and sex-matched normative population demonstrating a significant HrQoL burden exerted by POTS on a young, predominantly female population, including significant problems with usual activities, pain and discomfort, mobility, anxiety and depression, and self-care. Effective strategies to manage, improve, and support the reduced HrQoL in those with POTS are urgently needed.

## Supplementary Information

Below is the link to the electronic supplementary material.

Supplementary file1 (DOCX 25 KB)

## Data Availability

The data that support the findings of this study are not openly available due to reasons of sensitivity and confidentiality. These data are available from the corresponding author upon reasonable request.
