# Supplementary material for: Poor health-related quality of life in postural orthostatic tachycardia syndrome in comparison with a sex- and age-matched normative population
Source: Clin Auton Res. 2023 Jun 20;33(4):469–77. doi: 10.1007/s10286-023-00955-9 (PMC10439037; doi:10.1007/s10286-023-00955-9)
Supplement: Supplementary file 1 — Supplementary file1 (DOCX 25 KB) [file 10286_2023_955_MOESM1_ESM.docx]

**Supplementary File**

**Poor health-rated quality of life in postural orthostatic tachycardia syndrome**

**in comparison to a sex- and age-matched normative population**

Marie-Claire Seeley, MNurs;^1,2^ Celine Gallagher, PhD;^1,2^ Eric Ong, B HealthSci;^3^ Amy Langdon, B HealthSci;^3^ Jonathan Chieng,^1^ Danielle Bailey,^1^ Annabelle Dennis, BHumNut;^1^ Nikki McCaffrey, PhD;^4^ Dennis H Lau DH, MBBS, PhD^1,2,5^

*Short Title: Disutility in POTS*

**From:** ^1^Australian Dysautonomia and Arrhythmia Research Collaborative, The University of Adelaide, ^2^South Australian Health and Medical Research Institute, South Australia, Australia ^3^College of Medicine and Public Health, Flinders University, South Australia, Australia; ^4^Deakin Health Economics, Institute for Health Transformation, School of Health and Social Development, Faculty of Health, Deakin University, Victoria, Australia; & ^5^Department of Cardiology, Royal Adelaide Hospital, South Australia, Australia

**Correspondence:** Dennis H. Lau, MBBS, PhD; Department of Cardiology, Royal Adelaide Hospital, 1 Port Road, Adelaide, SA 5000, AUSTRALIA. Telephone: +61883175300; E-mail: [dennis.h.lau@adelaide.edu.au](mailto:dennis.h.lau@adelaide.edu.au)

| **Supplemental Table 1: Health-related quality of life - EQ-5D-5L sub-domains** | | | |
| --- | --- | --- | --- |
|  | **POTS** | **Normative** |  |
|  | n (%) | n (%) | ***P*-value** |
| ***Mobility*** |  |  |  |
| No problems | 57 (28) | 76 (88) | <.001 |
| Slight problems | 60 (30) | 13 (6) |  |
| Moderate problems | 60 (30) | 7 (4) |  |
| Severe problems | 25 (12) | 3 (1) |  |
| Unable to mobilize | 0 | 4 (1) |  |
|  |  |  |  |
| ***Self-Care*** |  |  |  |
| No problems | 94 (46) | 197 (97) | <.001 |
| Slight problems | 59 (29) | 5 (3) |  |
| Moderate problems | 33 (16) | 0 |  |
| Severe problems | 16 (8) | 0 |  |
| Unable to care for self | 0 | 0 |  |
|  |  |  |  |
| ***Usual activities*** |  |  |  |
| No problems | 16 (8) | 181 (90) | <.001 |
| Slight problems | 49 (24) | 13 (6) |  |
| Moderate problems | 62 (31) | 5 (3) |  |
| Severe problems | 49 (24) | 3 (1) |  |
| Unable to do usual activities | 26 (13) | 0 |  |
|  |  |  |  |
| ***Pain and discomfort*** |  |  | <.001 |
| No problems | 14 (7) | 124 (61) |  |
| Slight problems | 54 (27) | 58 (29) |  |
| Moderate problems | 83 (41) | 13 (6) |  |
| Severe problems | 39 (19) | 6 (3) |  |
| Extreme pain and discomfort | 11 (5) | 1 (.5) |  |
|  |  |  |  |
| ***Anxiety and depression*** |  |  |  |
| No problems | 59 (29) | 147 (73) | <.001 |
| Slight problems | 61 (30) | 39 (19) |  |
| Moderate problems | 62 (31) | 11(5) |  |
| Severe problems | 12 (6) | 3 (2) |  |
| Extreme anxiety and depression | 8 (4) | 2(1) |  |

|  | | | | | | | | | | | | | | | | | |
| --- | --- | --- | --- | --- | --- | --- | --- | --- | --- | --- | --- | --- | --- | --- | --- | --- | --- |
| **Supplemental Table 2: Median (IQR) utility and EQ-VAS scores between POTS and normative populations by age category** | | | | | | | | | | | | | | | | | |
|  | | **POTS** | | | | |  | | | **Normative population** | | | | | |  |  |
| ***Age Category*** | ***n*** | | *EQ-VAS* | | *Utility* | | | |  | | *EQ-VAS* | | *Utility* | |  |  |  |
| 16-24 years | 70 | | 40.5  (32) | | 0.65  (0.331) | | | |  | | 85***  (20) | | 0.95***  (10) | | | |  |
| 25-34 years | 68 | | 35  (32) | | 0.570  (0.37) | | | |  | | 85***  (19) | | 1.00***  (0.06) | | | |  |
| 35-44 years | 35 | | 40  (30) | | 0.63  (.27) | | |  | | 85***  (25) | | | | 0.94***  (0.11) | |  |  |
| 45-74 years | 29 | | 43  (20) | | 0.66  (0.183) | | |  | | 75***  (40) | | | | 0.86**  (0.32) | |  |  |
| Total | 202 | | 40  (30) | | 0.63  (0.317) | |  | | 85***  (25) | | | 0.95***  (0.10) | | | |  |  |
| *** P*<.01*; *** P*<.001 | | | |  | |  | |  | | | | | |  | | | |
